# Magnetodynamic properties of ultrathin films of Fe$_{\textbf{3}}$Sn$_{\textbf{2}}$-a topological kagome ferromagnet

**DOI:** 10.1038/s41598-024-53621-z

**Published:** 2024-02-12

**Authors:** Kacho Imtiyaz Ali Khan, Akash Kumar, Pankhuri Gupta, Ram Singh Yadav, Johan Åkerman, Pranaba Kishor Muduli

**Affiliations:** 1 Department of Physics, Indian Institute of Technology Delhi, Hauz Khas, New Delhi 110016 India; 2 Applied Spintronics Group, Department of Physics, University of Gothenburg, Gothenburg, 412 96 Sweden; 3 Center for Science and Innovation in Spintronics, Tohoku University, 2-1-1 Katahira, Aoba-ku, Sendai 980-8577 Japan; 4 Research Institute of Electrical Communication, Tohoku University, 2-1-1 Katahira, Aoba-ku, Sendai 980-8577 Japan

**Keywords:** Magnetic properties and materials, Electronic properties and materials, Spintronics

## Abstract

Fe$_3$Sn$_{2}$ is a topological kagome ferromagnet that possesses numerous Weyl points close to the Fermi energy, which can manifest various unique transport phenomena such as chiral anomaly, anomalous Hall effect, and giant magnetoresistance. However, the magnetodynamic properties of Fe$_3$Sn$_{2}$ have not yet been explored. Here, we report, for the first time, the measurements of the intrinsic Gilbert damping constant ($\alpha _\text{int}$), and the effective spin mixing conductance (g$_\text{eff}^{\uparrow \downarrow }$) of Pt/Fe$_3$Sn$_2$ bilayers for Fe$_3$Sn$_{2}$ thicknesses down to 2 nm, for which $\alpha _\text{int}$ is $(3.8 \pm 0.2) \times 10^{-2}$, and g$_\text{eff}^{\uparrow \downarrow }$ is $(11.7 \pm 0.6)~\text{nm}^{-2}$. The films have a high saturation magnetization, $M_\text{S}=620~\mathrm{emu~cm^{-3}}$, and large anomalous Hall coefficient, $R_\text{S}=4.6\times 10^{-10}~{\Omega ~\rm cm~G^{-1}}$. The large values of g$_\text{eff}^{\uparrow \downarrow }$, together with the topological properties of Fe$_3$Sn$_2$, make Fe$_3$Sn$_2$/Pt bilayers useful heterostructures for the study of topological spintronic devices.

## Introduction

The existence of strong electronic correlations, band topology, spin-orbit coupling, and magnetism in topological quantum materials holds great promise for future memory applications^1–7^. Weyl semimetals belong to a class of topological materials distinguished by the absence of either the crystal’s inversion symmetry or the time-reversal symmetry^8^. In Weyl semimetals, the opposite chirality of Weyl nodes can result in a non-trivial Berry phase^9–12^, which can influence the magneto-transport properties such as the anomalous Hall effect (AHE)^13–18^ and the anomalous Nernst effect (ANE)^19–21^. Recently, the kagome ferromagnet Fe$_3$Sn$_2$, belonging to the Fe$_m$Sn$_n$-family (*m* : *n* = 1:1, 3:2, 5:3), has emerged as a novel topological quantum material for spintronic devices, thanks to its rich non-trivial magnetic and topological properties^22–24^. Fe$_3$Sn$_{2}$, with a high Curie temperature $T_\text{C} = 657$ K^25^, which makes its Weyl nodes stable at room temperature^26^, has significant potential for applications in spintronics^27^, magnetic sensors^28^, and other areas of advanced electronics^29,30^. Fe$_3$Sn$_2$ possesses several other promising features, such as a large AHE^17^. It is also predicted that Fe$_3$Sn$_2$ can exhibit a fractional quantum Hall effect even at room temperature^31^. At temperature ($\sim 250$ K), the Fe$_3$Sn$_2$ shows the transition of spin re-orientation from the *c-*axis to the *ab-*plane^25,32,33^. Another interesting feature of Fe$_3$Sn$_2$ is the presence of a dispersionless flat band ($\sim 0.2$ eV below fermi level), and it is formed due to the destructive interference of the electron wavefunctions^34^. Furthermore, both numerical and experimental studies show the formation of magnetic skyrmions in Fe$_3$Sn$_2$, which is stabilized without requiring Dzyaloshinskii-Moriya interaction^30^.

**Figure 1:**
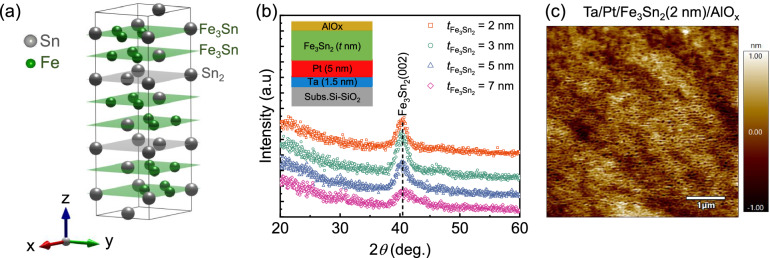
(**a**) Schematic of the unit cell of ferromagnet Fe$_3$Sn$_2$, where grey and green symbols denoting the tin (Sn) and iron (Fe) atoms, respectively. (**b**) Glancing incidence (GI) X-ray diffraction (XRD) spectra were obtained for the various thicknesses ($t_\mathrm{Fe_3Sn_2}$) of polycrystalline Fe$_3$Sn$_2$. The inset represents the schematic of Ta/Pt/Fe$_3$Sn$_2$/AlO$_x$ thin film stack. (**c**) Atomic force microscopy surface morphology of 2 nm-thick-Fe$_3$Sn$_2$ for a scan area of $5~\mathrm{\mu m}\times 5~\mathrm{\mu m}$.

As shown in Fig. 1a, the Fe$_{3}$Sn$_{2}$ crystal structure consists of the repeated stacking of two Fe$_{3}$Sn kagome lattices and one Sn$_2$ stanene lattice. In our previous study^35^, we investigated the impact of platinum (Pt) seed layer on the polycrystalline growth of ferromagnetic Fe$_3$Sn$_2$ thin films on complementary metal-oxide-semiconductor (CMOS)-compatible Si-based substrates, which are extremely useful for low-dissipation devices for industrial applications^36,37^. Furthermore, Lyalin et al. showed efficient spin-orbit torque effects in an epitaxial Fe$_3$Sn$_2$(0001)/Pt(111) bilayer system^27^ deposited using molecular beam epitaxy. However, the sputtered growth of ultrathin Fe$_3$Sn$_2$ films ($<10$ nm) and the characterization of their magneto-transport and magneto-dynamic properties have not yet been investigated, which is essential for the generation of pure spin current in such quantum material-based magnetic heterostructures.

In this work, we demonstrate sputter growth of high-quality polycrystalline Fe$_3$Sn$_2$ ultrathin films with very low interfacial/surface roughness ($<0.6$ nm), using a Ta/Pt seed layer on Si/SiO$_2$ substrates. Through magnetization and transport measurements, we show a large saturation magnetization, $M_\text{S}=620~\mathrm{emu~cm^{-3}}$, and a large anomalous Hall coefficient, $R_\text{S}=4.6\times 10^{-10}~{\Omega ~\rm cm~G^{-1}}$. Using broadband ferromagnetic resonance (FMR) measurements, we, for the first time, also extract the intrinsic Gilbert damping constant ($\alpha _\text{int}$), and the effective spin mixing conductance (g$_\text{eff}^{\uparrow \downarrow }$), for Fe$_3$Sn$_{2}$ thickness down to 2 nm, finding values of $\alpha _\text{int}=(3.8 \pm 0.2) \times 10^{-2}$, and g$_\text{eff}^{\uparrow \downarrow }=(11.7 \pm 0.6)~\text{nm}^{-2}$. The large values of g$_\text{eff}^{\uparrow \downarrow }$ make Pt an excellent spin current source for using Fe$_3$Sn$_2$ thin films in topological materials-based spintronic applications.

## Results and discussion

### Structural analysis

The inset of Fig. 1b shows a schematic of the Ta/Pt/Fe$_3$Sn$_2(t~\text{nm})$/AlO$_x$ thin film stack. First, a 1.5 nm-thin Ta seed layer was used to increase the adhesion between the Pt and the Si-SiO$_2$ substrate. The 5 nm-thick Pt seed layer was used both to promote the growth of the ferromagnetic phase of Fe$_3$Sn$_2$^27,35^ and to act as a spin sink and future source of spin currents, which will be discussed later. Fig. 1b shows the grazing incidence X-ray diffraction (GI-XRD) measurements performed for Si-SiO$_2$/Ta(1.5 nm)/Pt(5 nm)/Fe$_3$Sn$_2(t~\text{nm})$/AlO$_x$(3 nm) with an incidence angle 1$^\circ$ to characterize the structural properties. We observed a strong Bragg peak at 2$\theta ~=~40.6^\circ$, corresponding to the (002)-reflection of Fe$_3$Sn$_2$ for all thicknesses, indicating the formation of a [002]-oriented polycrystalline Fe$_{3}$Sn$_{2}$ ferromagnetic phase^28,35^. The thickness, density, and roughness of these Fe$_{3}$Sn$_{2}$ thin films were obtained by fitting X-ray reflectivity (XRR) measurements [supplementary, Fig. S1d] with the recursive theory of Parratt^38^. We found average interfacial roughness ($<0.6$ nm) for all the films, which indicates a smooth interface between each layer. AFM measurements also confirmed these roughness numbers. Figure 1c shows a $5~\mathrm{\mu m}\times 5~\mathrm{\mu m}$ AFM image of a 2 nm thick Fe$_{3}$Sn$_{2}$ film, yielding a root mean square roughness ($R_\text{rms}$) of about 0.3 nm, indicating a very smooth surface quality. The AFM $R_\text{rms}$ is b e l o w 0.6 nm for all other thicknesses. The thickness dependence of surface/interfacial roughness in these films is plotted in supplementary Fig. S1 and summarized in Table S1.

It is noteworthy that the measured roughness is substantially lower than the best literature values of about 0.8 nm^39^. The interfacial roughness plays a crucial role in the transfer of spin current in ferromagnet/heavy metal (FM/HM) heterostructures, where a large interfacial roughness or disorder can reduce the spin current via spin memory loss^40,41^. Therefore, high-quality ultra-thin films with low roughness are highly desirable.

### Magnetization and transport measurements

**Figure 2:**
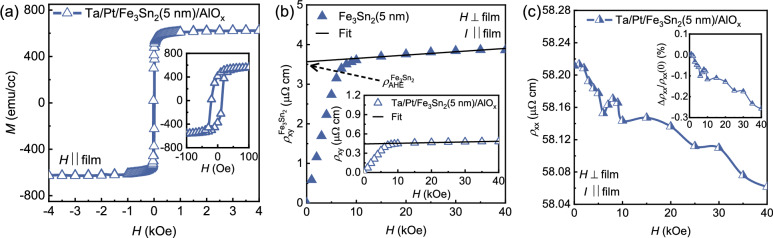
(**a**) In-plane magnetic hysteresis measurements (*M-H*) for 5 nm ultra-thin Fe$_{3}$Sn$_{2}$ film, inset represents the corresponding zoom scan. (**b**) The transverse Hall resistivity ($\rho _\text{xy}$) versus magnetic field for 5 nm ultra-thin Fe$_{3}$Sn$_{2}$ film when the external field is swept perpendicular to the film plane. The data in the inset with the open symbols indicates the measured $\rho _\text{xy}$, which includes the resistivity contribution from the seed layer, while the data in the main panel with the closed symbol indicates the $\rho _\text{xy}^\mathrm{Fe_{3}Sn_{2}}$ after correction for the resistivity of the seed layer in the Ta/Pt/Fe$_3$Sn$_2$/AlO$_x$ film stack. The black dashed arrow indicates the anomalous Hall resistivity ($\rho _{xy}^\mathrm{Fe_{3}Sn_{2}}$) for only Fe$_{3}$Sn$_{2}$. (**c**) The variation of longitudinal resistivity $\rho _{xx}$ for 5 nm ultra-thin Fe$_{3}$Sn$_{2}$ film when the external field is applied perpendicular to the film plane, inset represents the magnetoresistance (MR) calculated using $\Delta \rho _\text{xx}/\rho _\text{xx}(0)$. All measurements are performed at room temperature.

Figure 2a shows the magnetization (*M*) versus the in-plane external magnetic field (*H*) for a 5 nm Fe$_3$Sn$_2$ film. The high $M_\text{S}$ = 620 emu/cm$^{3}$ and low $H_\text{c}<$ 20 Oe confirm a soft ferromagnetic nature of the polycrystalline Fe$_3$Sn$_2$ films. The $M_\text{S}$ is comparable to that reported for epitaxial Fe$_3$Sn$_2$ films^21,39^ and bulk single crystals^16^.

In contrast to our earlier work on thicker Fe$_3$Sn$_2$ films^35^, additional care must be taken when extracting the longitudinal ($\rho _{xx}$) and transverse ($\rho _{xy}$) resistivities as the current distribution through the Ta/Pt seed layer must be considered. The total longitudinal resistivity of the entire film stack is found to be $113~{\mu \Omega ~\rm cm}$. We also measured $\rho _{xx}^\text{SL}$ of only the seed layer Ta/Pt, in control samples without Fe$_3$Sn$_2$, and found it to be $83~{\mu \Omega ~\rm cm}$, which corresponds to the longitudinal conductivity, $\sigma _{xx}^\text{SL}\sim 1.2\times 10^{4}~\mathrm{\Omega ^{-1}cm^{-1}}$. Using the parallel resistance model, the value $\rho _{xx}^\mathrm{Fe_{3}Sn_{2}}$ for only the Fe$_3$Sn$_2$ layer can be obtained using the following expression^21^:

1 $$\begin{aligned} \rho _{xx}^\mathrm{Fe_{3}Sn_{2}}= \frac{\rho _{xx}^\text{SL}~\rho _{xx}~t_\mathrm{Fe_{3}Sn_{2}}}{(t\cdot \rho _{xx}^\text{SL}) -(t_\text{SL}\cdot \rho _{xx})}, \end{aligned}$$

Here, $t_\mathrm{Fe_{3}Sn_{2}}$, $t_\text{SL}$ and *t* represent the thickness of the $\rm Fe_{3}Sn_{2}$ layer, seed (Ta/Pt) layer, and Ta/Pt/$\rm Fe_{3}Sn_{2}$ layer, respectively. Using Eq. (1), $\rho _{xx}^\mathrm{Fe_{3}Sn_{2}}$ is found to be $211~{\mu \Omega ~\rm cm}$ , which is comparable to that of epitaxial thin films ($202~{\mu \Omega ~\rm cm}$)^21^ and slightly higher than the bulk value of single crystals ($190~{\mu \Omega ~\rm cm}$)^16^.

In Fig. 2b and c, room temperature Hall and longitudinal measurements were performed using a direct current ($I=5$ mA) flowing parallel to the film plane while sweeping the external magnetic field ($H=\pm 40$ kOe) perpendicular to the film plane. To avoid voltage probe misalignment, we use the formulae $\rho _{xx}$ (*H*) = [$\rho _{xx}$ ($+H$) $+\rho _{xx}$ ($-H$)]/2 and $\rho _{xy}$ (*H*) = [$\rho _{xy}$ ($+H$) $-\rho _{xy}$ ($-H$)]/2, to extract the longitudinal resistivity ($\rho _{xx}$) and Hall resistivity ($\rho _{xy}$), respectively. To determine the $\rho _{xy}$, we use $\rho _{xy} = \rho _\text{OHE} + \rho _\text{AHE}$, where the first term represents the ordinary Hall resistivity ($\rho _\text{OHE}=R_{0}H$), and the second term represents the anomalous Hall resistivity ($\rho _\text{AHE}=R_\text{S}4\pi M_\text{eff}$). $R_0$ and $R_\text{S}$ represent the coefficients of ordinary and anomalous Hall resistivity, respectively^42^. $R_0$ is found to be $8.38\times 10^{-12}~{\Omega ~\rm cm/G}$, from which we determine the value of the charge carrier density $n=$ $0.74\times 10^{22}~\mathrm{cm^{-3}}$ at 300 K in Ta/Pt/Fe$_3$Sn$_2$(5 nm)/AlO$_x$. The positive sign of $R_0$ indicates that hole-like charge carriers dominate in Ta/Pt/Fe$_3$Sn$_2$(5 nm)/AlO$_x$ films, which is in agreement with previous reports^39^. Furthermore, we determine the carrier mobility $\mu ~=R_0/\rho _{xx}^\mathrm{Fe_{3}Sn_{2}}=$ $39.7~\mathrm{cm^{2}/V\cdot s}$ at 300 K, which is two orders of magnitude larger than earlier reported values [$\approx 0.08~\mathrm{cm^{2}/V\cdot s}$ for Fe$_3$Sn$_2$(10 nm)]^39^. The large $\mu$ might be due to the low effective mass of the hole carriers in the Fe$_3$Sn$_2$(5 nm) film, similar to the reported mobility for Weyl semimetal NbP ($\approx 160~\mathrm{cm^{2}/V\cdot s}$ at 300 K)^43^. In the inset of Fig. 2b, the measured transverse resistivity $\rho _{xy}$ of the complete film stack Ta/Pt/Fe$_3$Sn$_2$(5 nm)/AlO$_x$ is shown. Using a linear fit (black line) to $\rho _{xy}$ in the saturation region ($10~\text{kOe}<H<40~\text{kOe}$), and extrapolating to the *y-*axis, $\rho _\text{AHE}$ for the Ta/Pt/Fe$_3$Sn$_2(5~\text{nm})$/AlO$_x$ films stack is found to be $0.5~{\mu \Omega ~\rm cm}$. To determine the value of $\rho _{xy}^\mathrm{Fe_{3}Sn_{2}}$ for the Fe$_3$Sn$_2$ layer from the measured data for the complete film stack of Ta/Pt/Fe$_3$Sn$_2$/AlO$_x$, we use the expression^21^:

2 $$\begin{aligned} \rho _{xy}^\mathrm{Fe_{3}Sn_{2}} = \rho _{xy}\times \frac{\rho _{xx}^\mathrm{Fe_{3}Sn_{2}}}{\rho _{xx}}\left( 1+\frac{\rho _{xx}^\mathrm{Fe_{3}Sn_{2}}\times t_\text{SL}}{\rho _{xx}^\text{SL}\times t_\mathrm{Fe_{3}Sn_{2}}} \right) . \end{aligned}$$

As shown in Fig. 2b, the value of $\rho _\text{AHE}^\mathrm{Fe_{3}Sn_{2}}$ of only the Fe$_3$Sn$_2$ layer (denoted by a black dashed arrow) is extracted from the saturation region of $\rho _{xy}^\mathrm{Fe_{3}Sn_{2}}$ and found to be $3.56~{\mu \Omega ~\rm cm}$. This value for polycrystalline Fe$_3$Sn$_2$ ultrathin film is comparable to the *epitaxial* Fe$_3$Sn$_2$ thin film reported by D. Khadka et al.^21^. Using $M_\text{S}\approx 620~\mathrm{emu~cm^{-3}}$ from SQUID measurements, we also determine the coefficient ($R_\text{S}$) of the anomalous Hall resistivity for Ta/Pt/Fe$_3$Sn$_2$(5 nm)/AlO$_x$ film. The value of $R_\text{S}$ for Ta/Pt/Fe$_3$Sn$_2$(5 nm)/AlO$_x$ film is found to be $4.6\times 10^{-10}~{\Omega \mathrm {cm~G}^{-1}}$ at 300 K, which is comparable to our previous report on polycrystalline Fe$_3$Sn$_2$ thin films^35^ and two orders higher than conventional ferromagnets (Ni & Fe)^44,45^. Moreover, we determine the value of the anomalous Hall conductivity ($|\sigma _\text{AHE}^\mathrm{Fe_{3}Sn_{2}}|$) using the equation: $|\sigma _\text{AHE}^\mathrm{Fe_{3}Sn_{2}}|\approx (\rho _\text{AHE}^\mathrm{Fe_{3}Sn_{2}})/(\rho _{xx}^\mathrm{Fe_{3}Sn_{2}})^{2}$. The value of $|\sigma _\text{AHE}^\mathrm{Fe_{3}Sn_{2}}|$ is found to be $\approx 82~\mathrm{\Omega ^{-1}cm^{-1}}$ at 300 K. A large value of $R_\text{S}$ and $|\sigma _\text{AHE}^\mathrm{Fe_{3}Sn_{2}}|$ in Fe$_3$Sn$_2$film indicates an intrinsic band structure (Berry curvature) origin of the AHE^16,17,35^. These results indicate that the intrinsic transport properties, such as a large value of $R_\text{S}$ and a significant $|\sigma _\text{AHE}^\mathrm{Fe_{3}Sn_{2}}|$, remain intact even for ultra-low thicknesses of Fe$_3$Sn$_2$ films. In Fig. 2c, we have also plotted the variation of longitudinal resistivity ($\rho _{xx}$) versus external magnetic field for 5 nm ultra-thin Fe$_{3}$Sn$_{2}$ film. The inset of Fig. 2c represents the corresponding magnetoresistance (MR$=\Delta \rho _\text{xx}/\rho _\text{xx}(0)$) of Ta/Pt/Fe$_3$Sn$_2$(5 nm)/AlO$_x$ film. A negative change in MR in our thin films is caused due to the suppression of magnon at room temperature (300 K), consistent with the previous report on single crystal Fe$_3$Sn$_2$^16,22^.

### Ferromagnetic resonance measurement

**Figure 3:**
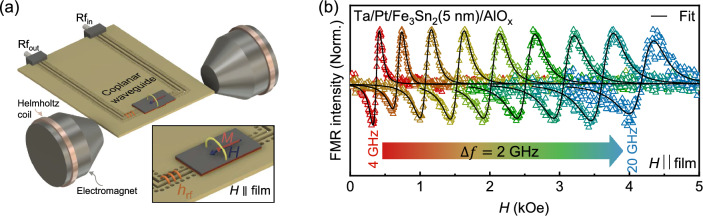
(**a**) The schematic of CPW-based FMR setup, inset shows the zoom image of the sample placed on top of CPW sweeping the external magnetic field *H* parallel to the film plane. (**b**) The frequency dependence of this FMR spectrum (open triangles) was obtained for Fe$_3$Sn$_2$(5 nm) and fitted (solid black line) with the sum of the derivative of the Lorentzian function.

**Figure 4:**
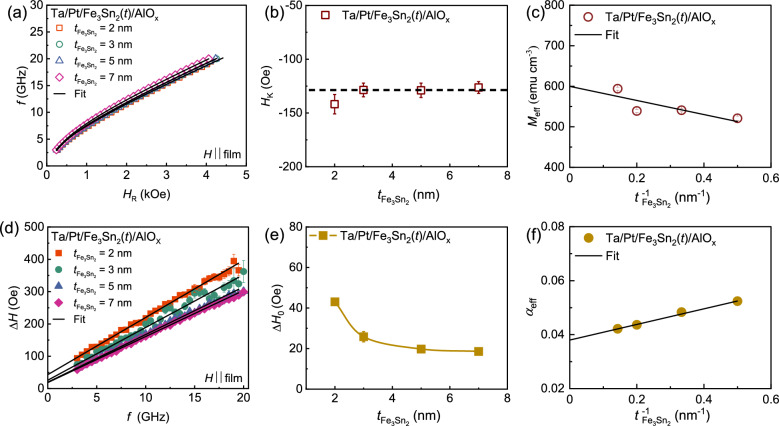
(**a**) Frequency (*f*) plotted as a function of resonance field $(H_\text{R})$ and fitted with Kittel Eq. (3). (**b**) The dependence of uniaxial anisotropy field $H_\text{K}$ over the thickness of Fe$_3$Sn$_{2}$ film, the dotted line represents the average value of $H_\text{K}$. (**c**) The extracted value of $M_\text{eff}$ is plotted over the inverse thickness ($t_\mathrm{Fe_{3}Sn_{2}}^{-1}$) of the ferromagnet and fitted with Eq. (4). (**d**) The variation of linewidth $(\Delta H)$ with frequency (*f*) and fitted with linewidth Eq. (5). (**e**) The variation of inhomogeneous linewidth $\Delta H_{0}$ plotted over the thickness of Fe$_3$Sn$_{2}$ film. (**f**) Effective damping constant ($\alpha _\text{eff}$) as a function of inverse thickness ($t_\mathrm{Fe_{3}Sn_{2}}^{-1}$) of ferromagnet together with the fit using Eq. (6). Here, the solid symbols and solid lines represent the experimental data and fit, respectively.

Figure 3a represents the schematic of a co-planar waveguide (CPW) based FMR setup with the film placed on top of it. Here, *H* is the external magnetic field swept parallel to the film plane and perpendicular to the rf excitation field ($h_\text{rf}$). The FMR setup details can be found in the Methods section. Fig. 3b shows FMR measurements for a Ta/Pt/Fe$_3$Sn$_2$(5 nm)/AlO$_x$ thin film. The frequency (*f*) dependent FMR spectra are shown at an interval of 2 GHz. The solid black lines are fits to derivatives of symmetric and asymmetric Lorentzian functions^46–49^. From these fits, we extract the resonance field ($H_\text{R}$) and linewidth ($\Delta H$) in the frequency range $4-20$ GHz. The variation of *f* is plotted as a function of $H_\text{R}$ in Fig. 4a for all Fe$_3$Sn$_2$ thicknesses, and then fitted to the Kittel formula^50^:

3 $$\begin{aligned} f=\frac{\gamma }{2\pi }\sqrt{(H_\text{R}+H_\text{K})(H_\text{R}+H_\text{K}+4\pi M_\text{eff})}, \end{aligned}$$

Here, $\gamma$ is the gyromagnetic ratio. $H_\text{R}$, $H_\text{K}$, and $M_\text{eff}$ are the resonance field, uniaxial anisotropy field, and the effective saturation magnetization of the ferromagnet. Using the value of $\gamma =185~\mathrm{GHz/T}$^27^ and fitting with Eq. (3) we extracted $H_\text{K}$ and $M_\text{eff}$ for different thickness of Fe$_3$Sn$_2$ [Fig. 4b and c]. In Fig. 4b, the average value of $|H_\text{K}|$ for Fe$_3$Sn$_2$(2-7 nm) films is found to be around 130 Oe. Furthermore, the uniaxial anisotropy constant ($K_{u}$) of Fe$_3$Sn$_2$ film is determine by: $K_{u}=H_\text{K}M_\text{S}/2$. The value of $K_{u}$ is found to be $9.3\times 10^{4}~\mathrm{erg~cm^{-3}}$, which is one order lower than bulk single crystal^51^. In Fig. 4c, the behavior of $M_\text{eff}$ over the thickness of Fe$_3$Sn$_2$ is plotted and fitted with the equation;

4 $$\begin{aligned} M_\text{eff}~=~M_\text{S}-\frac{2K_\text{S}}{\mu _{0}M_\text{S}}\times {t_\mathrm{Fe_{3}Sn_{2}}^{-1}}, \end{aligned}$$

Here, $\mu _{0}$ is the permeability constant of free space. $M_\text{S}$ and $K_\text{S}$ are the saturation magnetization and surface anisotropy constant, respectively. From the fitting, the values of $M_\text{S}$ and $K_\text{S}$ are found to be $(599\pm 29)~\mathrm{emu~cm^{-3}}$ and $(0.29\pm 0.02)~\mathrm{erg~cm^{-2}}$, respectively. It is noteworthy that we found a good agreement between the values of $M_\text{S}$ obtained from the FMR technique and the SQUID data.

The $\Delta H$ versus *f* for all the thicknesses is plotted (solid open symbol) in Fig. 4(d) and fitted with the expression^52–55^:

5 $$\begin{aligned} \Delta H= \Delta H_\mathrm{{0}}+\frac{2\pi \alpha _\text{eff}}{\gamma }f, \end{aligned}$$

Here, the first term, $\Delta H_\mathrm{{0}}$ denotes the inhomogeneous broadening, which largely depends on the quality of the sample. The second term indicates the effective damping ($\alpha _\text{eff}$). In Fig. 4d, from fits of $\Delta H$ versus *f* with the Eq. (5) for various thicknesses, we extracted $\Delta H_{0}$ and $\alpha _\text{eff}$. The value of the inhomogeneous broadening, $\Delta H_\mathrm{{0}}$ is found to be less than 40 Oe for all films [as shown in Fig. 4e]. Here, we found a monotonic increase in $\alpha _\text{eff}$ for the thickness of ferromagnet Fe$_3$Sn$_{2}$. Figure 4f shows the value of $\alpha _\text{eff}$ with the inverse of ferromagnetic thickness ($t_\mathrm{Fe_{3}Sn_{2}}^{-1}$). The behavior was fitted with^56,57^:

6 $$\begin{aligned} \alpha _\text{eff} = \alpha _\text{int}+\text{g}^{\uparrow \downarrow }_\text{eff}\frac{\gamma \hslash }{4\pi M_\text{S} }\times t_\mathrm{Fe_{3}Sn_{2}}^{-1}, \end{aligned}$$

where $\alpha _\text{int}$ represent the intrinsic Gilbert damping constant of the ferromagnet Fe$_3$Sn$_{2}$, while and g$^\mathrm{\uparrow \downarrow }_\text{eff}$ represent the effective spin mixing conductance of Pt/Fe$_3$Sn$_{2}$ system. From the fitting, we found $\alpha _\text{int}$ to be around $(3.8\pm 0.2)\times 10^{-2}$ and g$^\mathrm{\uparrow \downarrow }_\text{eff}$ to be $(11.7\pm 0.6)~\mathrm{nm^{-2}}$. The $\alpha _\text{int}$ depends on both spin-orbit coupling as well as the phase lag between the distortions of the Fermi surface and the precessing magnetization. The intrinsic mechanism of Gilbert damping is commonly ascribed to spin-orbit coupling through two potential mechanisms: interband and intraband scattering^58,59^. In the interband scattering mechanism, the magnetization dynamics can generate electron-hole pairs across different bands. This leads to a Gilbert damping effect that scales with the resistivity^60,61^. Conversely, in the intraband scattering scenario, electron-hole pairs are generated within the same electronic band, resulting in a Gilbert damping effect that scales with the conductivity^62,63^. Our value of $\alpha _\text{int}$ is relatively larger compared to transition metal thin films, and since the resistivity of Fe$_3$Sn$_{2}$ is found to be larger, we speculate that the mechanism of intrinsic damping in our polycrystalline Fe$_3$Sn$_2$ films is “resistivity-like”. However, more studies (e.g., temperature dependence) are needed to determine the mechanism of intrinsic damping in Fe$_3$Sn$_{2}$. The order of g$^\mathrm{\uparrow \downarrow }_\text{eff}$ for Fe$_3$Sn$_{2}$/Pt is almost comparable to other Pt-based FM heterostructures^48,64,65^, indicating that ferromagnet Fe$_3$Sn$_{2}$ can also be used as an effective spin current source. Hence, a large value of spin mixing conductance in Fe$_3$Sn$_{2}$/Pt bilayer system, together with its exotic magneto-transport properties, can be beneficial for memory-based device applications.

## Conclusion

In summary, we demonstrate the growth of ultra-thin polycrystalline phase of Fe$_3$Sn$_{2}$ films with varying thicknesses (2-7 nm). The XRD, XRR, and AFM results show high-quality films with low surface/interfacial roughness. The magneto-static and magneto-transport results suggest the formation of the ferromagnetic phase and the intrinsic AHE nature of Fe$_3$Sn$_{2}$ films, respectively. Here, we report the first measurements of the intrinsic Gilbert damping constant ($\alpha _\text{int}$), and effective spin mixing conductance (g$_\text{eff}^{\uparrow \downarrow }$) in Fe$_3$Sn$_2$ films. The extracted value of $\alpha _\text{int}$, and g$_\text{eff}^{\uparrow \downarrow }$ is found to be $(3.8 \pm 0.2) \times 10^{-2}$, and $(11.7 \pm 0.6)~\text{nm}^{-2}$, respectively. A large value of g$_\text{eff}^\mathrm{\uparrow \downarrow }$ obtained from FMR measurements suggest ferromagnet Fe$_3$Sn$_{2}$ can also be a potential material to generate pure spin current. These results promote the inexpensive and widely used sputter material growth of such quantum materials.

## Methods

### Sample preparation

The ultrathin films of Fe$_3$Sn$_2(t~\text{nm})$ with varying thicknesses (*t* = 2, 3, 5, and 7) on Si-SiO$_2$ substrate were deposited using RF magnetron sputtering^35^ at room temperature. An optimized low growth rate of $\approx$ 0.2  $\mathring{\text{A}}~\mathrm s^{-1}$ was used for better control over the ultra-low thickness of Fe$_3$Sn$_2$ films. The base pressure of the sputtering chamber was better than 6.7$\times 10^{-8}$ mbar, while the working pressure was maintained at 2.7$\times 10^{-3}$ mbar. These thin films were post-annealed *in-situ* at $500~^{\circ }$C for 1 hour to improve the crystallinity. A 3 nm aluminum (Al) layer was capped on all films to protect these samples from oxidation.

### Sample characterization

The structural properties of these films were analyzed with the help of the X-ray diffraction (XRD) technique using a PANalytical X’Pert diffractometer with Cu-$K_\alpha$ radiation ($\lambda = 1.5418~\mathring{\text{A}}$). The elemental and compositional analyses of these films were determined with the help of the electronic probe microscopy analysis (EPMA) technique. We determine the composition of Fe and Sn to be 61 at.% and 39 at.% in all the samples. The average surface roughness and topography of these films were obtained using the atomic force microscopy (AFM) technique in tapping mode (Asylum Research, MFP-3D system). The thickness, roughness, and density of these Fe$_3$Sn$_2$ films were measured using the X-ray reflectivity (XRR) measurement technique. The static magnetization measurements were carried out using the magnetic property magnetic system (MPMS) with a superconducting quantum interference device (SQUID) using Quantum Design Inc. The magneto-transport properties were measured using the physical property measurement system (PPMS) technique from Quantum Design Inc. (Evercool-II). We employed four-terminal sensing techniques: linear contact geometry for determining longitudinal resistivity and Hall contact geometry for the transverse resistivity. One pair of contact electrodes is used to supply the DC current in the sample, while the other pair of contact electrodes perpendicular (parallel) to the current direction is used for sensing the transverse (longitudinal) voltage. The magneto-dynamic measurements are performed using NanOsc PhaseFMR-40 FMR setup in the 4-20 GHz frequency range. The instrument used field modulation (AC field of 1 Oe peak to peak) for a higher signal-to-noise ratio (using Helmholtz coils with 490 Hz reference frequency). The measurements are performed with an RF power of 12.5-17.6 dBm (varying for different frequency ranges).

### Supplementary Information

Supplementary Information.

## Data Availability

The datasets used and analysed during the current study available from the corresponding author on reasonable request.

## References

[1] Tokura, Y., Kawasaki, M. & Nagaosa, N. Emergent functions of quantum materials. *Nat. Phys.* **13**, 1056–1068 (2017).

[2] Han, W., Otani, Y. & Maekawa, S. Quantum materials for spin and charge conversion. *npj Quantum Mater.* **3**, 27 (2018).

[3] Shao, Q. *et al.* Roadmap of spin-orbit torques. *IEEE Trans. Magn.* **57**, 1–39 (2021). 10.48550/arXiv.2104.11459 PMC10091395 37057056

[4] Wang, Y. & Yang, H. Spin-orbit torques based on topological materials. *Acc. Mater. Res.* **3**, 1061–1072 (2022).

[5] Hasan, M. Z. & Kane, C. L. Colloquium: Topological insulators. *Rev. Mod. Phys.* **82**, 3045 (2010).

[6] Chowdhury, N. *et al.* Kagome magnets: The emerging materials for spintronic memories. *Proc. Natl. Acad. Sci. India Sect. A* **93**, 477–495 (2023).

[7] Kumar, A. *et al.* Interfacial Origin of Unconventional Spin-Orbit Torque in Py/-IrMn. *Adv. Quantum Technol.* 2300092 (2023).

[8] Armitage, N., Mele, E. & Vishwanath, A. Weyl and Dirac semimetals in three-dimensional solids. *Rev. Mod. Phys.* **90**, 015001 (2018).

[9] Lv, B. *et al.* Observation of Weyl nodes in TaAs. *Nat. Phys.* **11**, 724–727 (2015).

[10] Xu, S.-Y. *et al.* Discovery of a Weyl fermion state with Fermi arcs in niobium arsenide. *Nat. Phys.* **11**, 748–754 (2015).

[11] Weng, H., Fang, C., Fang, Z., Bernevig, B. A. & Dai, X. Weyl semimetal phase in noncentrosymmetric transition-metal monophosphides. *Phys. Rev. X* **5**, 011029 (2015).

[12] Soluyanov, A. A. *et al.* Type-II weyl semimetals. *Nature* **527**, 495–498 (2015). 26607545 10.1038/nature15768

[13] Taguchi, Y., Oohara, Y., Yoshizawa, H., Nagaosa, N. & Tokura, Y. Spin chirality, Berry phase, and anomalous Hall effect in a frustrated ferromagnet. *Science* **291**, 2573–2576 (2001). 11283363 10.1126/science.1058161

[14] Nagaosa, N., Sinova, J., Onoda, S., MacDonald, A. H. & Ong, N. P. Anomalous Hall effect. *Rev. Mod. Phys.* **82**, 1539 (2010).

[15] Nayak, A. K. *et al.* Large anomalous Hall effect driven by a nonvanishing Berry curvature in the noncolinear antiferromagnet MnGe. *Sci. Adv.* **2**, e1501870 (2016). 27152355 10.1126/sciadv.1501870 PMC4846447

[16] Ye, L. *et al.* Massive Dirac fermions in a ferromagnetic kagome metal. *Nature* **555**, 638–642 (2018). 29555992 10.1038/nature25987

[17] Kida, T. *et al.* The giant anomalous Hall effect in the ferromagnet FeSn-a frustrated kagome metal. *J. Phys.: Condens. Matter* **23**, 112205 (2011). 21358031 10.1088/0953-8984/23/11/112205

[18] Wang, Q. *et al.* Large intrinsic anomalous Hall effect in half-metallic ferromagnet CoSnS with magnetic Weyl fermions. *Nat. Commun.* **9**, 3681 (2018). 30206233 10.1038/s41467-018-06088-2 PMC6134149

[19] Chen, T. *et al.* Large anomalous Nernst effect and nodal plane in an iron-based kagome ferromagnet. *Sci. Adv.* **8**, eabk1480 (2022). 35030028 10.1126/sciadv.abk1480 PMC8759748

[20] Miyasato, T. *et al.* Crossover behavior of the anomalous Hall effect and anomalous Nernst effect in itinerant ferromagnets. *Phys. Rev. Lett.* **99**, 086602 (2007). 17930968 10.1103/PhysRevLett.99.086602

[21] Khadka, D. *et al.* Anomalous Hall and Nernst effects in epitaxial films of topological kagome magnet FeSn. *Phys. Rev. Mater.* **4**, 084203 (2020).

[22] Li, H. *et al.* Large topological Hall effect in a geometrically frustrated kagome magnet FeSn. *Appl. Phys. Lett.* **114**, 192408 (2019).

[23] Le Caer, G., Malaman, B., Haggstrom, L. & Ericsson, T. Magnetic properties of FeSn. III. A Sn Mossbauer study. *J. Phys. Condens. Matter.* **9**, 1905 (1979).

[24] Ren, Z. *et al.* Plethora of tunable Weyl fermions in kagome magnet FeSn thin films. *npj Quantum Mater.* **7**, 109 (2022).

[25] Le Caër, G., Malaman, B. & Roques, B. Mossbauer effect study of FeSn. *J. Phys. F Met. Phys.* **8**, 323 (1978).

[26] Yao, M. *et al.* Switchable Weyl nodes in topological Kagome ferromagnet FeSn. Preprint at arXiv:1810.01514 (2018).

[27] Lyalin, I., Cheng, S. & Kawakami, R. K. Spin-orbit torque in bilayers of kagome ferromagnet FeSn and Pt. *Nano Lett.* **21**, 6975–6982 (2021). 34380320 10.1021/acs.nanolett.1c02270

[28] Satake, Y., Fujiwara, K., Shiogai, J., Seki, T. & Tsukazaki, A. Fe-Sn nanocrystalline films for flexible magnetic sensors with high thermal stability. *Sci. Rep.* **9**, 1–7 (2019). 30824854 10.1038/s41598-019-39817-8 PMC6397158

[29] Du, Q. *et al.* Room-Temperature Skyrmion Thermopower in FeSn. *Adv. Quantum Technol.* **3**, 2000058 (2020).

[30] Hou, Z. *et al.* Observation of various and spontaneous magnetic skyrmionic bubbles at room temperature in a frustrated kagome magnet with uniaxial magnetic anisotropy. *Adv. Mater.* **29**, 1701144 (2017). 10.1002/adma.201701144 28589629

[31] Tang, E., Mei, J.-W. & Wen, X.-G. High-temperature fractional quantum Hall states. *Phys. Rev. Lett.* **106**, 236802 (2011). 21770532 10.1103/PhysRevLett.106.236802

[32] Fenner, L., Dee, A. & Wills, A. Non-collinearity and spin frustration in the itinerant kagome ferromagnet FeSn. *J. Phys. Condens. Matter* **21**, 452202 (2009). 21694002 10.1088/0953-8984/21/45/452202

[33] Heritage, K. *et al.* Images of a first-order spin-reorientation phase transition in a metallic kagome ferromagnet. *Adv. Funct. Mater.* **30**, 1909163 (2020).

[34] Lin, Z. *et al.* Flatbands and emergent ferromagnetic ordering in FeSn kagome lattices. *Phys. Rev. Lett.* **121**, 096401 (2018). 30230862 10.1103/PhysRevLett.121.096401

[35] Khan, K. I. A. *et al.* Intrinsic anomalous Hall effect in thin films of topological kagome ferromagnet FeSn. *Nanoscale* **14**, 8484–8492 (2022). 35662312 10.1039/d2nr00443g

[36] Feng, Y. P. *et al.* Prospects of spintronics based on 2D materials. *Wiley Interdiscip. Rev. Comput. Mol. Sci.* **7**, e1313 (2017).

[37] Barla, P., Joshi, V. K. & Bhat, S. Spintronic devices: A promising alternative to CMOS devices. *J. Comput. Electron.* **20**, 805–837 (2021).

[38] Parratt, L. G. Surface studies of solids by total reflection of X-rays. *Phys. Rev.* **95**, 359 (1954).

[39] Zhang, D., Hou, Z. & Mi, W. Anomalous and topological Hall effects of ferromagnetic FeSn epitaxial films with kagome lattice. *Appl. Phys. Lett.* **120**, 232401 (2022).

[40] Gupta, K., Wesselink, R. J., Liu, R., Yuan, Z. & Kelly, P. J. Disorder dependence of interface spin memory loss. *Phys. Rev. Lett.* **124**, 087702 (2020). 32167325 10.1103/PhysRevLett.124.087702

[41] Belashchenko, K. D., Kovalev, A. A. & van Schilfgaarde, M. Theory of spin loss at metallic interfaces. *Phys. Rev. Lett.* **117**, 207204 (2016). 27886511 10.1103/PhysRevLett.117.207204

[42] Hurd, C. M. *Hall Effect in Metals and Alloys* (Plenum Press, 1972).

[43] Shekhar, C. *et al.* Extremely large magnetoresistance and ultrahigh mobility in the topological Weyl semimetal candidate NbP. *Nat. Phys.* **11**, 645–649 (2015).

[44] Volkenshtein, N. & Fedorov, G. Temperature dependence of the Hall effect of pure ferromagnets. *Sov. Phys. JETP* **11**, 48–50 (1960).

[45] Kaul, S. N. Anomalous Hall effect in nickel and nickel-rich nickel-copper alloys. *Phys. Rev. B* **20**, 5122 (1979).

[46] Woltersdorf, G. *Spin-Pumping and Two-Magnon Scattering in Magnetic Multilayers* (Simon Fraser University, 2004).

[47] Zhang, W., Han, W., Jiang, X., Yang, S.-H. & SP Parkin, S. Role of transparency of platinum-ferromagnet interfaces in determining the intrinsic magnitude of the spin Hall effect. *Nat. Phys.* **11**, 496–502 (2015).

[48] Kumar, A. *et al.* Influence of annealing on spin pumping in sputtered deposited Co/Pt bilayer thin films. *Phys. B Cond. Matt.* **570**, 254–258 (2019).

[49] Kumar, A., Bansal, R., Chaudhary, S. & Muduli, P. K. Large spin current generation by the spin hall effect in mixed crystalline phase Ta thin films. *Phys. Rev. B* **98**, 104403 (2018).

[50] Kittel, C. On the theory of ferromagnetic resonance absorption. *Phys. Rev.* **73**, 155 (1948).

[51] Tang, J. *et al.* Target bubbles in FeSn nanodisks at zero magnetic field. *ACS Nano* **14**, 10986–10992 (2020). 32806036 10.1021/acsnano.0c04036

[52] Rossing, T. D. Resonance linewidth and anisotropy variation in thin films. *J. Appl. Phys.* **34**, 995–995 (1963).

[53] Heinrich, B., Cochran, J. & Hasegawa, R. FMR linebroadening in metals due to two-magnon scattering. *J. Appl. Phys.* **57**, 3690–3692 (1985).

[54] Celinski, Z. & Heinrich, B. Ferromagnetic resonance linewidth of Fe ultrathin films grown on a bcc Cu substrate. *J. Appl. Phys.* **70**, 5935–5937 (1991).

[55] McMichael, R. D., Twisselmann, D. & Kunz, A. Localized ferromagnetic resonance in inhomogeneous thin films. *Phys. Rev. Lett.* **90**, 227601 (2003). 12857340 10.1103/PhysRevLett.90.227601

[56] Bangar, H. *et al.* Large spin hall conductivity in epitaxial thin films of kagome antiferromagnet MnSn at room temperature. *Adv. Quant. Tech.* **6**(1), 2200115 (2022).

[57] Khan, K. I. A., Gupta, P., Agarwal, R., Chowdhury, N. & Muduli, P. K. Comparative study of spin pumping in epitaxial-and polycrystalline-NiO/NiFe. *SPIN* (2023).

[58] Heinrich, B. *Ultrathin Magnetic Structures I: An Introduction to the Electronic, Magnetic and Structural Properties* (Springer-Verlag, Springer Science & Business Media, 2005).

[59] Mewes, C. K. & Mewes, T. *Relaxation in Magnetic Materials for Spintronics* (Pan Stanford, 2015).

[60] Ma, X. *et al.* Role of antisite disorder on intrinsic Gilbert damping in FePt films. *Phys. Rev. B* **91**, 014438 (2015).

[61] Heinrich, B. & Frait, Z. Temperature dependence of the FMR linewidth of iron single-crystal platelets. *Phys. Stat. Solidi (b)* **16**, K11–K14 (1966).

[62] Kamberskỳ, V. On the Landau-Lifshitz relaxation in ferromagnetic metals. *Can. J. Phys.* **48**, 2906–2911 (1970).

[63] Khodadadi, B. *et al.* Conductivitylike Gilbert damping due to intraband scattering in epitaxial iron. *Phys. Rev. Lett.* **124**, 157201 (2020). 32357022 10.1103/PhysRevLett.124.157201

[64] Mosendz, O. *et al.* Quantifying spin Hall angles from spin pumping: Experiments and theory. *Phys. Rev. Lett.* **104**, 046601 (2010). 20366725 10.1103/PhysRevLett.104.046601

[65] Ando, K. *et al.* Inverse spin-Hall effect induced by spin pumping in metallic system. *J. Appl. Phys.* **109**, 10 (2011).

