# Supplementary material for: Magnetodynamic properties of ultrathin films of Fe3Sn2-a topological kagome ferromagnet
Source: Sci Rep. 2024 Feb 12;14:3487. doi: 10.1038/s41598-024-53621-z (PMC11269729; doi:10.1038/s41598-024-53621-z)
Supplement: Supplementary file 1 — Supplementary Information. [file 41598_2024_53621_MOESM1_ESM.pdf]

Magnetodynamic properties of ultrathin films of the Fe_3Sn_2-a topological kagome ferromagnet

Kacho Imtiyaz Ali Khan[1], Akash Kumar[2,3,4], Pankhuri Gupta[1], Ram Singh Yadav[1] Johan Åkerman[2,3,4*] and Pranaba Kishor Muduli[1*]

[1]Department of Physics, Indian Institute of Technology Delhi, Hauz Khas, New Delhi, 110016, India

[2]Applied Spintronics Group, Department of Physics, University of Gothenburg, Gothenburg 412 96, Sweden

[3]Research Institute of Electrical Communication, Tohoku University, 2-1-1 Katahira, Aoba-ku, Sendai, 980-8577, Japan

[4]Center for Science and Innovation in Spintronics, Tohoku University, 2-1-1 Katahira, Aoba-ku, Sendai, 980-8577, Japan

*To whom correspondence should be addressed; E-mails: muduli@physics.iitd.ac.in, johan.akerman@physics.gu.se

E-mail:

Surface/interfacial roughness analysis.

In Fig. S1(e), the results show similar variations of interfacial roughness and surface roughness obtained from the XRR technique and the AFM technique, respectively. The obtained values of interfacial roughness and surface roughness for all the samples were summarized in Table S1. Therefore, a low surface/interfacial roughness (< 0.6 nm) indicates the high quality of ultra-thin films, which will be beneficial for the spin current flowing through these interfaces in quantum material-based magnetic heterostructures.

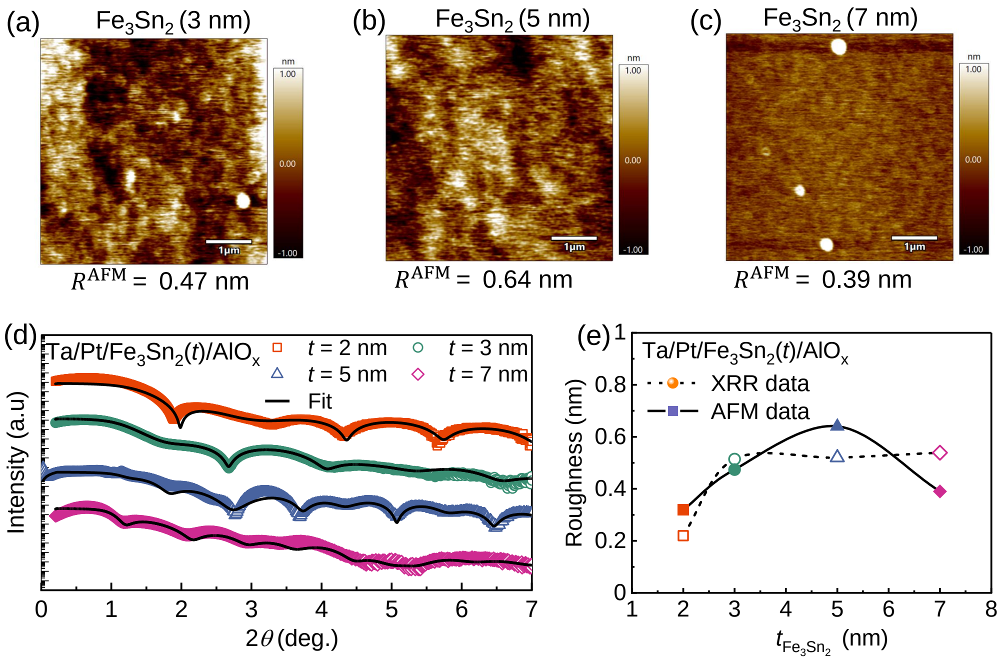

Figure S1: (a)-(c) The surface morphology of Fe_3Sn_2(t nm) (t =3, 5, 7) thin film obtained from the atomic force microscopy technique. (d) The solid symbols represent the measured X-ray reflectivity (XRR) spectra for all thicknesses of Fe_3Sn_2 thin film, and the solid lines represent the corresponding fits. (e) The plot indicates the variation in interfacial roughness data obtained from the XRR technique and the variation in surface roughness data obtained from the AFM technique.

Table S1: The densities, thicknesses, and interfacial/Surface roughnesses of the Ta/Pt/Fe_3Sn_2(t nm)/AlOx film stack.

Sample stack	Elements	Density (g/cc)	Thickness, t (nm)	Interfacial roughness, R^{XRR} (nm)	Surface roughness, R^{AFM} (nm)
Ta/Pt/Fe_3Sn_2(2)/AlOx	Ta	15.58	1.38	0.21	
	Pt	23.87	4.59	0.58	
	**Fe_3Sn_2**	**7.83**	**1.75**	**0.22**	0.32
	AlOx	2.28	2.69	1.35	
Ta/Pt/Fe_3Sn_2(3)/AlOx	Ta	9.34	1.09	0.36	
	Pt	18.59	3.98	0.55	
	**Fe_3Sn_2**	**10.08**	**2.82**	**0.52**	0.47
	AlOx	1.98	2.78	0.51	
Ta/Pt/Fe_3Sn_2(5)/AlOx	Ta	18.10	1.80	0.16	
	Pt	25.70	5.36	0.25	
	**Fe_3Sn_2**	**9.35**	**5.28**	**0.52**	0.64
	AlOx	2.25	2.52	0.67	
Ta/Pt/Fe_3Sn_2(7)/AlOx	Ta	16.28	2.10	0.11	
	Pt	18.75	3.54	0.44	
	**Fe_3Sn_2**	**7.51**	**6.76**	**0.54**	0.39
	AlOx	2.62	3.21	1.19	
